# Dietary Curcumin Supplementation Could Improve Muscle Quality, Antioxidant Enzyme Activities and the Gut Microbiota Structure of *Pelodiscus sinensis*

**DOI:** 10.3390/ani13162626

**Published:** 2023-08-14

**Authors:** Jia-Yuan Jiang, Hua Wen, Ming Jiang, Juan Tian, Li-Xue Dong, Ze-Chao Shi, Tong Zhou, Xing Lu, Hong-Wei Liang

**Affiliations:** 1 Yangtze River Fisheries Research Institute, Chinese Academy of Fishery Sciences, Wuhan 430223, China; jiang9902022@163.com (J.-Y.J.); wenhua.hb@163.com (H.W.); jiangming@yfi.ac.cn (M.J.); tianjuan0303@163.com (J.T.); dlx@yfi.ac.cn (L.-X.D.); szc@yfi.ac.cn (Z.-C.S.); zhoutong@yfi.ac.cn (T.Z.); 2 College of Fisheries and Life Science, Shanghai Ocean University, Shanghai 201306, China

**Keywords:** *Pelodiscus sinensis*, reptile, dietary curcumin, antioxidant, hepatic metabolism, gut microbiome, gene expression

## Abstract

**Simple Summary:**

Curcumin (CM) is a hydrophobic polyphenolic compound derived from the rhizome of turmeric (*Curcuma longa* Linn.). It has been proven to have antioxidant, anti-inflammatory, and nutritional metabolic regulatory functions in mammals. Currently, little is known about its effects on reptiles. In this study, dietary supplementation of 2–4 g/kg curcumin improved the antioxidant enzyme activities and muscle quality of Chinese soft-shelled turtles (*Pelodiscus sinensis*). Moreover, dietary curcumin supplementation increased the abundance of antioxidant bacteria (*Lactobacillus* and *Flavobacterium*) in the gut of turtles and affected hepatic metabolism-related pathways, reducing the crude lipid content of the body. These data could guide curcumin application to promote the health status of turtles.

**Abstract:**

This experiment aimed to assess the impact of different dietary curcumin (CM) levels on growth, muscle quality, serum-biochemical parameters, antioxidant-enzyme activities, gut microbiome, and liver transcriptome in Chinese soft-shelled turtles (*Pelodiscus sinensis*). Five experimental diets were formulated to include graded levels of curcumin at 0 (control, CM0), 0.5 (CM0.5), 1 (CM1), 2 (CM2) and 4 g/kg (CM4). Each diet was randomly distributed to quadruplicate groups of turtles (164.33 ± 5.5 g) for 6 weeks. Our findings indicated that dietary curcumin supplementation did not have a significant influence on growth performance (*p* > 0.05); however, it significantly improved the muscular texture profiles (*p* < 0.05). Serum total superoxide dismutase (SOD), liver catalase (CAT), and total antioxidant capacity (T-AOC) activities increased significantly as dietary curcumin levels rose from 0.5 to 4 g/kg (*p* < 0.05). Dietary curcumin supplementation improved gut microbiota composition, as evidenced by an increase in the proportion of dominant bacteria such as *Lactobacillus* and *Flavobacterium*. Liver transcriptome analysis revealed that curcumin altered metabolic pathways in the liver. In conclusion, based on the evaluation of the activities of SOD in serum and CAT in liver under current experimental design, it was determined that the appropriate dietary curcumin supplementation for Chinese soft-shelled turtles is approximately 3.9 g/kg.

## 1. Introduction

The Chinese soft-shelled turtle, scientifically known as *Pelodiscus sinensis*, is an ancient reptile that has gained significant importance in aquaculture due to its high nutritional and economic value [1]. It is also known that the turtle has been used in traditional Chinese medicine (TCM) to address human health problems including inflammation, cancer, hypertension, menopause, diabetes, and anemia [2]. According to statistics from the China Fishery Statistical Yearbook of Agriculture (2022), the production of Chinese soft-shelled turtles in 2021 exceeded 364,870 tons [3]. In China, the cultivation of Chinese soft-shelled turtles primarily relies on high-density farming within the greenhouse. However, this intensive culture pattern inevitably caused water quality deterioration and widespread diseases [4]. As the market demand and large-scale farming for this species increase, numerous diseases in Chinese soft-shelled turtle breeding are on the rise [5,6]. Furthermore, consumers generally have a lower acceptance of the meat quality of farmed aquatic animals compared to wild aquatic animals [7]. Generally, for evaluation of fillet freshness, hardness reflects the internal binding force that maintains the compact shape of the flesh fillet, while springiness refers to the ability of the sample to regain its initial shape after removing the deforming force. Gumminess (g) indicates the force required to break down a meat sample for swallowing (hardness × cohesiveness). Chewiness is defined as the combination of hardness, cohesiveness, and springiness [8]. Therefore, enhancing health and flesh quality in turtle cultivation is of utmost importance.

Curcumin (CM) is one of the most common active polyphenols extracted from turmeric, which is a spice harvested from *Curcuma longa*, a plant of the ginger family [9]. In mammals, CM has been demonstrated to exert excellent effects such as cholesterol-lowering [10], anti-diabetic [11], anti-oxidant [12], anti-tumor [13], anti-microbial, appetite-increasing [14], and immunomodulatory [15]. Importantly, CM can protect the organism from reactive oxygen species (ROS) stress or other free radical-induced DNA damage, thus decreasing the risk of tumor formation. This function is due to its antioxidative mechanism, including metal chelation, radical neutralization, and hydrogen peroxide elimination [16]. Because of its antioxidant and anti-inflammatory properties documented in an array of scientific investigations, CM is regarded as a propitious supplement in aquaculture. For example, Xavier et al. [17] reported that dietary CM supplementation can elevate the antioxidant capacity and growth of Senegalese sole postlarvae (*Solea senegalensis*). Other researchers indicated that dietary CM administration could improve the immune response and disease resistance of Nile tilapia (*Oreochromis niloticus*) [18] and rainbow trout (*Oncorhynchus mykiss*) [19]. Nonetheless, the application of CM in reptilian species remains relatively under-explored.

Dietary intervention serves as an efficient method for promoting growth and health in aquatic animals. In this sense, we explored whether dietary CM could improve the growth performance, nutritional composition, antioxidant ability, and gut health of Chinese soft-shelled turtles. These data would provide a theoretical basis for the rational application of CM in the diet of Chinese soft-shelled turtles.

## 2. Materials and Methods

### 2.1. Experimental Diet Preparation

The curcumin powder (purity ≥ 98%) was supplied by a commercial company. CM was supplemented separately into an isonitrogenous (approximately 420 g/kg crude protein) and isolipidic (approximately 91 g/kg crude lipid) basal diet to obtain five diets with a level of 0 (control, CM0), 0.5 (CM0.5), 1 (CM1), 2 (CM2) and 4 g/kg (CM4). The formulation of the diets was according to a previous report [1]. The formulation and proximate compositions of experimental diets are presented in Table S1. Using high-pressure liquid chromatography, the actual concentration of CM in five diets was 0, 0.487, 0.998, 1.989 and 3.398 g/kg, respectively.

All feed ingredients were ground and sieved through a 40 μm mesh before being mixed. The resulting feed was then wet-extruded through a pelletizer (230, Hangzhou SaiXu Food Machinery Co., Ltd., Hangzhou, China) to produce 2 mm diameter pellets. These pellets were sealed in plastic bags after drying and preserved at −20 °C for further utilization.

### 2.2. Experimental Turtles and Feeding Management

The animal experiment was conducted in accordance with the animal care protocols approved by the Animal Welfare Committee of Yangtze River Fisheries Research Institute, Chinese Academy of Fishery Sciences. Chinese soft-shelled turtles were procured from the Anhui Xijia Agricultural Development Co., Ltd. (Bengbu, China). The feeding experiment took place at the Liangzihu experimental base of the Yangtze River Fisheries Research Institute (E’zhou, China). All turtles were provided with a basal diet to facilitate acclimatization to the experimental conditions.

Following a 2-week acclimation period, 160 healthy turtles (initial body weight: 164.33 ± 5.5 g, approximately 1 year old) were randomly distributed into 20 rectangular plastic tanks (L 90 cm × W 60 cm × H 35 cm). Each group contained four experimental tanks with eight turtles per tank, and each diet was randomly assigned to four replicates. To prevent injuries resulting from aggressive behavior, every tank was furnished with a food table and three pieces of mesh for turtles to climb and inhabit. Water depth was adjusted to facilitate respiration for individual turtles. Turtles were hand-fed at 3% of their body weight at 07:30, 12:30 and 17:30 for six weeks. This feeding ratio approximated the maximum daily consumption for turtles based on their feed consumption within 30 min. Approximately 30% of the water was exchanged in each tank every morning, and uneaten feed or fecal matter was siphoned out to maintain the water quality. Turtles were weighed collectively once every two weeks, with feed amounts adjusted accordingly. Throughout the feeding period, the water temperature was maintained at 27 ± 3 °C and pH at 7.1–7.4. The experiment was conducted under a natural photoperiod, and mortality was recorded daily.

### 2.3. Sample Collection

After the trial was finished, the turtles were fasted for 24 h before being sampled. The number of turtles in each tank was recorded and their weight and length were measured individually to calculate the weight gain. Twelve turtles from each treatment (three per tank) were randomly selected for muscle composition analysis and texture profile analysis (TPA). Blood samples were obtained from twelve randomly chosen turtles in each treatment group. Blood samples were collected from the head vein of anesthetized turtles, centrifuged at 1000× *g* for 15 min at 4 °C, and the supernatant was stored at −80 °C for blood chemistry analysis. The turtles were subsequently dissected on ice to isolate liver and intestine tissues. Each treatment collected three tubes of liver and midgut tissue, with each tube containing tissue from two turtles. Liver tissue samples from each treatment group were collected for enzymatic activity determination, while the remaining liver samples were utilized for transcriptome sequencing and gene-expression analysis. Intestine tissues were harvested for gut-microbiome assessment. The collected tissues were immediately snap frozen in liquid nitrogen and stored at −80 °C until further analysis.

### 2.4. Muscle Composition Analysis and Texture Profile Analysis

The proximate composition of the experimental diets and muscle samples was assessed according to the standard method of the Association of Official Analytical Chemists [20]. Crude protein content was ascertained by detecting nitrogen content (N × 6.25) using an automatic Kjeldahl nitrogen analyzer (Kjeldahl K360, BUCHI, Flawil, Switzerland). Crude lipid content was examined through petroleum ether extraction using a Soxhlet apparatus. Ash content was determined by calcinating samples at 550 °C for 8 h in a muffle furnace. Moisture content was assessed by drying samples in an oven at 105 °C until a constant weight was achieved.

Muscles without skin were separated from the turtle limbs and cut into 1 cm^3^ sections (1 cm × 1 cm × 1 cm) for texture profile analysis (TPA). TPA was conducted using a texture analyzer (Stable Micro Systems, Ltd., London, UK) to investigate the specified parameters, encompassing hardness, springiness, chewiness, cohesiveness, and resilience. TPA, a double compression cycle test, was executed up to 30% compression of the initial thickness employing a cylindrical aluminum probe. The probe was affixed to the analyzer apparatus with its longitudinal side oriented perpendicularly to the surface of the turtle’s muscle tissue. The analysis was performed at a velocity of 2 mm s^−1^.

### 2.5. Serum and Liver Biochemical Analyses

The serum specimens were gently defrosted at 4 °C, followed by evaluation utilizing an automated biochemistry analyzer (Sysmex-800, Sysmex Corporation, Kobe, Japan). Multiple parameters, encompassing total protein (TP), albumin (ALB), triacylglycerol (TG), cholesterol (TCHO), glucose (GLU), alkaline phosphatase (ALP) activity, and aspartate transaminase (AST) activity, were examined via the automated biochemistry analyzer (Sysmex-800, Sysmex Corporation, Kobe, Japan) and commercial diagnostic reagent kits (Sysmex Wuxi Co., Ltd., Wuxi, China), adhering to the manufacturer’s protocols.

Antioxidant enzymes in the liver or serum, such as superoxide dismutase (SOD), catalase (CAT), and total antioxidant capacity (T-AOC), were identified using commercial reagent kits procured from the Nanjing Jian Cheng Bioengineering Institute (Nanjing, China), in accordance with previously delineated methodologies [21].

### 2.6. Gut Microbiota Analysis

The gut samples from CM0, CM1, and CM4 groups were dissected for DNA extraction. After the determination of DNA quality and concentration, an equivalent of 2 ng/μL DNA for each sample was utilized for 16S rRNA sequencing, as reported in our previous study [22]. The primers 338F 5′-ACTCCTACGGGAGGCAGCA-3′ and 806R 5′-GGACTACHVGGGTWTCTAAT-3′ were used to amplify the V3-V4 regions of the bacteria 16S ribosomal. For analysis of the gut-microbiome bioinformatics, raw sequences were performed with the quantitative insights into microbial ecology (QIIME) tool with a slight modification according to the official tutorials (https://docs.qiime2.org/2022.2/tutorials/, accessed on 12 March 2023). The following databases were used as operational taxonomic units (OTUs) taxonomic status identification: Greengenes (Release 13.8, http://greengenes.secondgenome.com, accessed on 12 March 2023), Silva (Release 115, http://www.arbsilva.de, accessed on 12 March 2023), UNITE (Release 5.0, https://unite.ut.ee/, accessed on 12 March 2023). Briefly, any OTUs with an abundance of less than 0.001% of the total sequencing of the whole sample were removed. Sequences were assigned to OTUs at 97% similarity. One-way analysis of variance (ANOVA) was used to analyze the significance of the differences between the different groups, and *p* < 0.05 was considered as significant difference.

### 2.7. RNA Sequencing and Bioinformatics Analysis

Total RNA was isolated from hepatic tissue samples in groups CM0, CM1, and CM4 employing the TRIzol reagent (Invitrogen, Carlsbad, CA, USA), adhering to the manufacturer’s instructions. Subsequent to extraction, RNA concentrations were ascertained utilizing a NanoDrop2000 spectrophotometer (Thermo Scientific, Washington, WA, USA), while RNA integrity was assessed via an Agilent 2100 bioanalyzer. A quantity of 3 micrograms (μg) of RNA served as input material for the preparation of RNA samples. As previously delineated [23], sequencing libraries were constructed employing the Illumina TruSeq™ RNA Sample Preparation Kit. Triplicate biological replicates were executed for each group independently. Ultimately, the sequencing libraries underwent sequencing on an Illumina HiSeq2000 platform, facilitated by Shanghai Personal Biotechnology Co., Ltd. (http://www.personalbio.cn, accessed on 12 March 2023).

The raw data underwent processing, which entailed the removal of adapter sequences, empty reads, and sequences of low quality (Q < 30). Subsequently, clean reads were aligned to the reference genome of *Pelodiscus sinensis* (https://www.ncbi.nlm.nih.gov/genome/?term=pelodiscus+sinensis, accessed on 15 March 2023, PRJNA221645, Pelsin_1.0) using Hisat2 software (v.2.0.4) [24]. Gene-expression levels were quantified as fragments per kilobase per million fragments mapped (FPKM). Differentially expressed genes (DEGs) were discerned between pairs of groups, employing criteria of *p*-value < 0.05 and |log2(fold change)| > 1, subsequently categorizing them into upregulated and downregulated transcript lists. Blast2GO (https://www.blast2go.com/, accessed on 15 March 2023) was utilized to obtain biological process (BP), molecular function (MF), and cellular component (CC) terms. GO terms with a false discovery rate (FDR) < 0.05 were deemed significantly enriched. The Kyoto Encyclopedia of Genes and Genomes (KEGG) was employed to assign and predict putative functional roles and biological pathways correlated with DEGs.

### 2.8. Quantitative Real-Time PCR Assay (qRT-PCR)

A qRT-PCR assay was performed as described in the previous literature [25] to validate the results of RNA-seq. Total RNA extraction from liver-tissue samples in CM0, CM1, and CM4 groups was executed utilizing TRIzol reagent (Life Technologies, Carlsbad, CA, USA). To eliminate genomic DNA contamination, RNA samples were subjected to DNase I digestion (Takara Biotechnology, Dalian, China). Subsequently, RNA integrity and quality assessment were conducted via 1.0% agarose electrophoresis and spectrophotometric analysis. Each RNA sample underwent reverse transcription to synthesize first-strand cDNA using the Prime-Script™ RT reagent kit (Takara Biotechnology).

The qRT-PCR assays were performed employing an ABI 7500 Real-Time PCR System in a 20-µL reaction volume, which comprised 10 µL of SYBR Premix Ex Taq II (Takara Biotechnology), 0.4 µL of ROX Reference Dye II (Takara), and 6.8 µL of RNase-free water. The thermocycling parameters included an initial denaturation at 95 °C for 30 s, followed by 40 cycles of 95 °C for 5 s, and 60 °C for 30 s. All reactions were executed in triplicate. Primer sequences were designed using Primer Premier 6.0 and displayed in Table S2; β-actin was selected as an internal gene. The reaction specificity was confirmed by observing a single peak at the expected Tm on the melting curve. The expression level of the target gene relative to the internal gene (β-actin) was calculated according to the 2^−ΔΔCt^ method [26].

### 2.9. Statistical Analysis

The results from all experiments are expressed as mean values accompanied by their respective standard deviations (SD). Differences among dietary groups were analyzed using the SPSS 26.0 software package (Chicago, IL, USA). Before performing statistical evaluations, the data were checked for homogeneity of variances and normal distribution. ANOVA and Tukey’s multiple-comparison tests were utilized to identify significant differences at a threshold of *p* < 0.05.

## 3. Results

### 3.1. Growth Performance

The growth performance is shown in Table 1. There were no significant differences in final body weight (FBW), weight gain (WG) and feed conversion ratio (FCR) among the five groups (*p* > 0.05).

### 3.2. Muscle-Proximate Composition and Texture Profile

The muscle-proximate composition of Chinese soft-shelled turtles is presented in Table 2. With the increment in dietary CM level from 0.5 to 4 g/kg, crude protein content significantly increased while crude lipid showed a decreased tendency (*p* < 0.05). Turtles in the CM4 group had the highest crude protein and the lowest crude lipid content, respectively. However, CM supplementation had no significant influence on muscle moisture and ash levels (*p* > 0.05).

Table 3 presents the texture profiles of the muscle samples. Compared to the CM0 group, both CM1 and CM2 groups exhibited a significant increase in hardness and chewiness (*p* < 0.05). The CM4 group demonstrated higher hardness and gumminess compared to the CM0 group (*p* < 0.05). No significant differences were observed in the springiness, cohesiveness, and resilience of the muscle samples (*p* > 0.05).

### 3.3. Serum Biochemistry Indexes

Table S3 exhibits the serum biochemical indices of Chinese soft-shelled turtles. CM supplementation in diet did not show significant effects on serum glucose (GLU) and albumin (ALB) levels (*p* > 0.05). Nevertheless, the total protein (TP) content in the CM0.5 group was significantly elevated compared to the other four groups (*p* < 0.05). The contents of triglyceride (TG) and total cholesterol (TCHO) were significantly elevated with increasing CM levels from 0.5 to 4 g/kg, and the highest values were all detected in the CM4 group (*p* < 0.05). In contrast, both alkaline phosphatase (ALP) and aspartate transaminase (AST) activities were significantly decreased in the CM4 group compared to the CM0 group (*p* < 0.05).

### 3.4. Antioxidant Enzyme Activities in Serum and Liver

**Figure 1:**
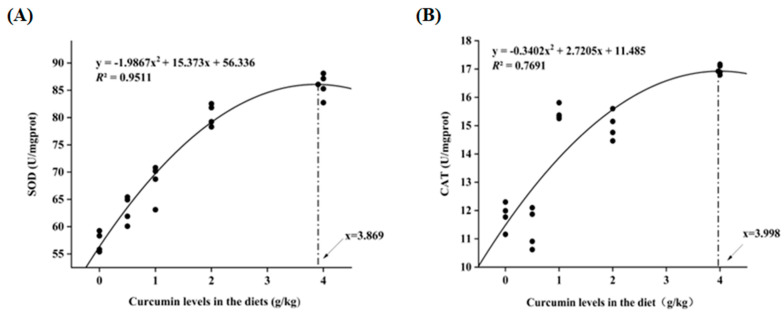
(**A**) Regression analysis between dietary CM supplementation levels and activity of SOD; (**B**) Regression analysis between dietary CM supplementation levels and activity of CAT. Each black dots represents a piece of data.

As depicted in Table 4, a significant elevation was observed in the activities of serum total superoxide dismutase (SOD), hepatic catalase (CAT), and total antioxidant capacity (T-AOC) concomitant with the escalating concentrations of dietary CM (*p* < 0.05). Their values reached the highest in CM4 group. Quadratic regression analysis of SOD and CAT provided an estimate of 3.869 g/kg and 3.998 g/kg of dietary CM for maximum antioxidant capacity (Figure 1A,B).

### 3.5. Gut Microbial Diversity Analysis

Approximately 1,880,977 raw reads were obtained from CM0, CM1 and CM4 groups. After removal of chimeras and short sequences, an average of 66,985 clean reads were produced for each sample. Utilizing the UPARSE clustering approach, a cumulative sum of 1453 operational taxonomic units (OTUs) was derived from the clean tags, as delineated in Table S4.

**Figure 2:**
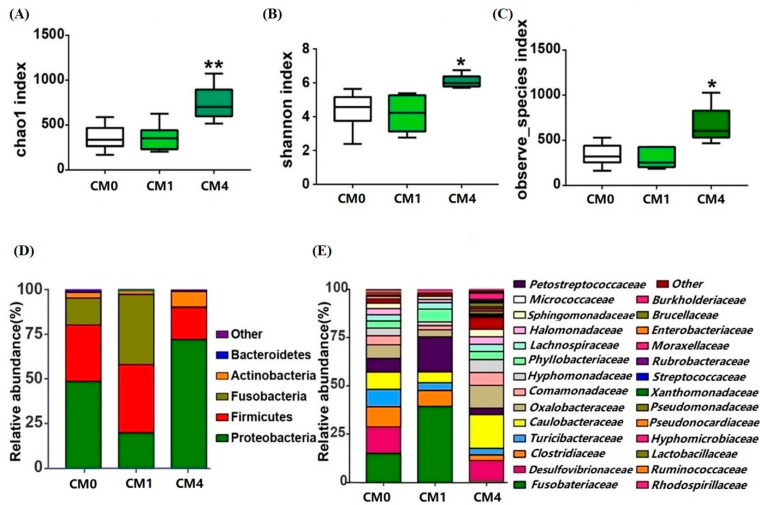
Gut microbiota variations among CM0, CM1 and CM4 groups. (**A**) Chao-1; (**B**) Shannon; (**C**) Observed_species; (**D**) Microbial composition at the phylum level by a stacked bar plot; (**E**) Microbial composition at the family level by a stacked bar plot. * *p* < 0.05 and ** *p* < 0.01 vs. CM0 group.

**Figure 3:**
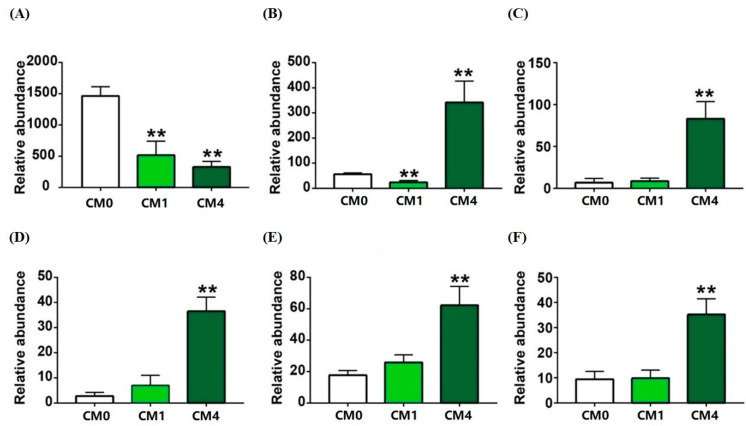
Significantly changed genera among CM0, CM1 and CM4 groups. (**A**) *Clostridium*; (**B**) *Pseudomonas*; (**C**) *Stenotrophomonas*; (**D**) *Flavobacterium*; (**E**) *Lactobacillus*; (**F**) *Blautia*. ** *p* < 0.01 vs. CM0 group.

As depicted in Figure 2A–C, a drastic clustering of gut microbiota between CM0 and CM4 group showed an obvious distinction of Chao1, Shannon and Observed species. At the phylum level, a diminution in the relative abundance of Proteobacteria was discerned within the CM1 group, whereas a decline in Firmicutes was evident in the CM4 group (Figure 2D). At the family level, an increased abundance of Fusobacteriaceae and Caulobacteraceae was detected in the CM1 and CM4 groups, respectively (Figure 2E). In comparison with the CM0 group, a considerable diminution in the abundance of Clostridium was distinctly observed in both CM1 and CM4 groups (Figure 3A). However, the richness of *Pseudomonas*, *Stenotrophomonas*, *Flavobacterium*, *Lactobacillus* and *Blautia* was significantly increased in the CM4 group (*p* < 0.05) (Figure 3B–F).

Moreover, five upregulated pathways (including 6-hydroxymethyl-dihydropterin diphosphate biosynthesis I, adenosylcobalamin salvage from cobinamide II, adenosylcobalamin biosynthesis from cobyrinate a, c-diamide I, tetrapyrrole biosynthesis II (from glycine), pentose phosphate pathway (non-oxidative branch)) and five downregulated pathways (such as L-methionine biosynthesis I, superpathway of pyrimidine nucleobases salvage, superpathway of S-adenosyl-L-methionine biosynthesis, superpathway of L-methionine biosynthesis (transsulfuration), L-isoleucine biosynthesis III) were enriched in the CM0 and CM1 groups (Figure S1A). Nine upregulated pathways (including superpathway of D-glucarate and D-galactarate degradation, D-galactarate degradation I, D-glucarate degradation I, L-arginine degradation (AST pathway), superpathway of ornithine degradation, glucose degradation (oxidative), glucose and glucose-1-phosphate degradation, L-histidine degradation II, superpathway of L-alanine biosynthesis) and seven downregulated pathways (such as L-glutamate degradation V (via hydroxyglutarate), mannan degradation, succinate fermentation to butanoate, galactose degradation I (LeIoir pathway), sucrose degradation III (sucrose invertase), adenosylcobalamin salvage from cobinamide II, teichoic acid (poly glycerol) biosynthesis) were determined in the CM0 and CM4 groups (Figure S1B).

### 3.6. Liver Transcriptomic Changes

**Figure 4:**
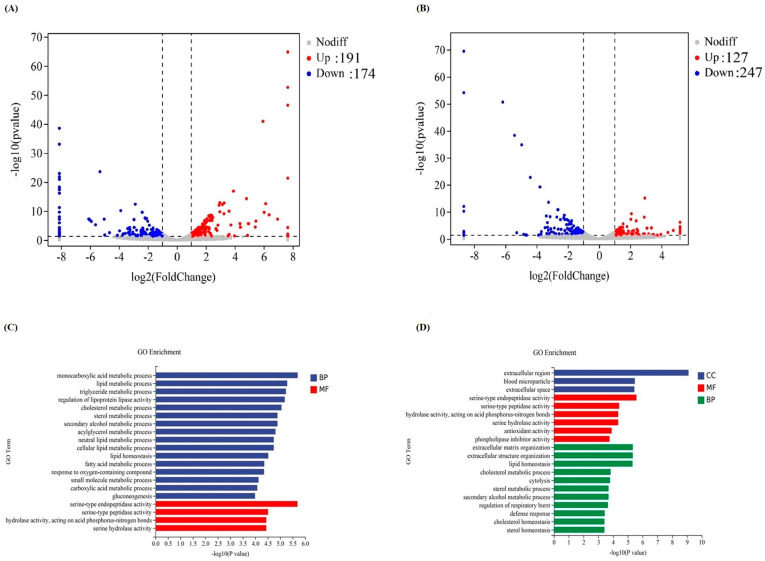
Global changes of transcriptome-sequencing results. (**A**) Volcano plots of differentially expressed genes with CM0 vs. CM1. (**B**) Volcano plots of differentially expressed genes with CM0 vs. CM4. Downregulated genes are shown with blue dots (*p* < 0.05 and log2Foldchange < −1) and upregulated genes with red (*p* < 0.05 and log2Foldchange > 1). Genes with gray dots did not show differential expression. Gene ontology (GO) classification of the significantly differential genes (*p* < 0.05). The results are summarized in three main GO categories: biological process (BP), cellular component (CC), and molecular function (MF). (**C**) GO enrichment analysis of DEGs in CM0 vs. CM1. (**D**) GO enrichment analysis of DEGs in CM0 vs. CM4. The x-axis indicates the gene quantity, and the y-axis indicates the gene functional category. The categories are presented in different colors.

The liver transcriptome analysis among CM0, CM1 and CM4 groups was performed to identify the gene transcriptional expressions. Upon eliminating inferior data, a sum of 75,940,858 unblemished reads was procured. A total of 365 distinct differentially expressed genes (DEGs) were discerned in the CM0 vs. CM1 comparison group, encompassing 191 upregulated and 174 downregulated genes. The DEG profile in the CM0 vs. CM4 comparison group ascertained 127 upregulated and 247 downregulated genes, correspondingly (Figure 4A–B). Gene ontology (GO) examination revealed that these DEGs predominantly coalesced into three classifications, comprising biological process (BP), cellular component (CC), and molecular function (MF) (Figure 4C–D). The DEGs in the CM0 vs. CM1 comparison group chiefly concentrated in GO terms incorporating monocarboxylic acid metabolic process, lipid metabolic process, and serine-type endopeptidase activity. In the CM0 vs. CM4 comparison group, the five most highly enriched GO terms included extracellular region, serine-type endopeptidase activity, extracellular matrix organization, extracellular structure organization, and lipid homeostasis. KEGG enrichment analysis revealed that within both the CM0 vs. CM1 and CM0 vs. CM4 comparison groups, the up-regulated genes were principally engaged in metabolism-related pathways, including the PPAR signaling pathway, FoxO signaling pathway, Adipocytokine signaling pathway, and Ferroptosis and Fatty acid degradation. Conversely, the down-regulated genes participated in processes such as Steroid biosynthesis, Phenylalanine, tyrosine and tryptophan biosynthesis, as well as ECM-receptor interaction (Figure S2A–D).

### 3.7. Gene Expression

**Figure 5:**
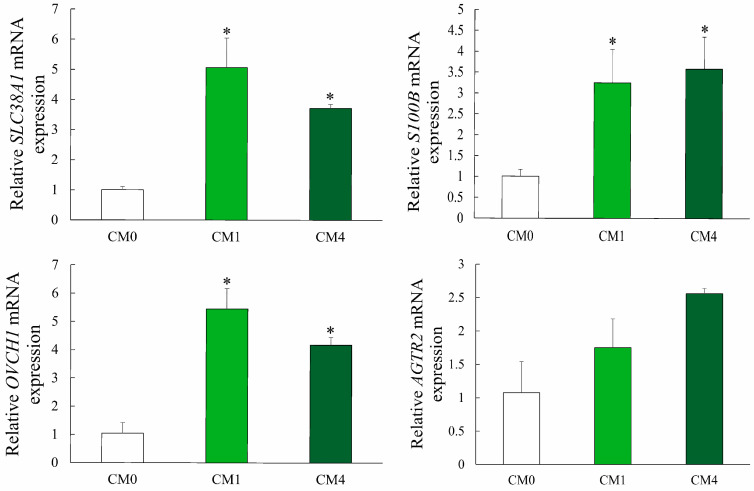
Gene-relative expression in response to different dietary CM treatments as detected by qPCR. Data are shown as means ± SD (standard deviation). * *p* < 0.05 vs. CM0 group.

As delineated in Table S2 and Figure 5, the upregulated expression of Solute Carrier Family 38 Member 1 (*SLC38A1*), S100 Calcium Binding Protein B (*S100B*), Ovochymase 1 (*OVCH1*) and Angiotensin II Receptor Type 2 (*AGTR2*) genes was observed in the liver of turtles fed with 1 g/kg and 4 g/kg CM-enriched diets.

## 4. Discussion

In the present study, dietary supplementation with CM showed no significant improvement in the growth of Chinese soft-shelled turtles. This result is similar to previous research conducted by Xavier et al. [17] and Chen et al. [27], which demonstrated that the addition of CM in diet had no significant effects on growth and feed utilization. However, other research on crucian carp (*Carassius auratus*) [28], rainbow trout (*Oncorhynchus mykiss*) [18] and Nile tilapia (*Oreochromis niloticus*) [29] indicated that supplementary CM extracts in diet could increase the weight gain (WG) and survival rate. The discrepancy may be related to the aquatic species, sizes, feeding management, and breeding environment (such as water temperature and dissolved oxygen). Moreover, the administration of CM at lower levels in diet did not significantly influence the feed-conversion ratio (FCR). This observation could be ascribed to the accelerated metabolism and clearance of CM within the animal organism, leading to diminished bioavailability. Previous studies have indicated that encapsulated formulations can enhance the biodistribution and bioavailability of CM [30]. The molecular mechanism underlying the impact of CM on soft-shelled turtles warrants further investigation.

With the increase of the CM level from 0.5 g/kg to 4 g/kg, there was a significant increase in crude protein content within the muscle composition, concomitant with a decrease in crude lipid content (*p* < 0.05). Furthermore, the genes related to adipose tissue (such as *SLC38A1* and *S100B*) were significantly upregulated in the liver. The obtained results were consistent with the early findings on mammals, which suggested that a supplement of CM can reduce fat deposition in adipocytes [31]. This phenomenon could be linked to the inherent characteristics of CM, which encompass the inhibition of angiogenesis and the reduction in both the quantity and content of adipocytes. Additionally, CM suppresses the differentiation of adipocyte precursor cells, consequently diminishing the overall lipid content within the organism [31,32]. As an important physical characteristic of muscle tissue, texture is one of the most-used indicators to evaluate the meat quality for aquatic products. In this study, the values of muscular hardness and gumminess were significantly higher in turtles fed the 4 g/kg CM diet. This improvement might be due to the high crude protein content and low lipid content, with beneficial effects on the growth of the muscle microstructure such as myofibrillar protein, matrix protein and muscle fiber. Similar effects on poultry have been also demonstrated in duck and broiler chicken [33,34].

Aspartate transaminase (AST) is an important transaminase that reflects the health status of mitochondria and the cytoplasm of hepatocytes. In our experiment, dietary CM decreased serum AST activity, and the lowest value was detected in the CM4 group (*p* < 0.05). This observation concurs with the findings reported by Abbas et al. [35], who demonstrated that the incorporation of CM in the diet significantly diminished serum ALT and AST activities in catfish (*Clarias gariepinus*). It can be explained that the molecular structure with diketone and phenolic hydroxyl groups in CM can effectively remove oxidative free radicals, exert an anti-inflammatory biological activity, and consequently protect tissues from stress damage [36,37]. Furthermore, the experimental group exhibited a significant elevation in serum TCHO and TG levels compared to the control group. Correspondingly, the *AGTR2* and *OVCH1* genes related to blood lipids were upregulated [38]. Previous studies have identified a positive and significant association between plasma CM concentration and serum cholesterol [39]. CM has been documented to stimulate the expression of ABCG1 in humans at a physiological concentration approximating 2 μmol/L, consequently increasing low abundance of high-density lipoproteins-(HDL) dependent lipid efflux, and plasma HDL cholesterol level [40].

Curcumin exhibits indirect antioxidant functions by enhancing superoxide dismutase (SOD) and plasma catalase (CAT) activities, as reported in prior literature [41,42]. In the present investigation, the activities of serum SOD, hepatic CAT, and T-AOC were significantly amplified by CM supplementation (*p* < 0.05). This effect may be associated with the chemical structure of CM, which features phenolic and methoxy groups. The interaction between these two groups can remove oxygen free radicals, inhibit lipid peroxidation, and further improve the antioxidant capacity of the body [43,44]. Similar results were obtained in common carp (*Cyprinus carpio*) and grass carp [45,46,47].

In aquaculture, bacterial diversity is reported to be linked with pathogenesis [48,49], and α-diversity is a potential biomarker for fish health [50]. Herein, dietary CM-level at 4 g/kg caused significant alteration in the bacterial α-diversity, suggesting it can improve the intestinal microbiome diversity of the soft-shelled turtle. The principal microorganisms influenced by CM, as evidenced in prior research, encompass Proteobacteria, Firmicutes, Actinobacteria, and Fusobacteria [51,52]. In addition, dietary CM treatment also increased the abundance of *Pseudomonas* and *Stenotrophomonas* genus, which are identified as the main protease-producing bacteria [53,54]. Notably, the increased richness of *Lactobacillus* and *Flavobacterium* were detected in the CM4 group. These two bacteria have also been shown to significantly promote disease resistance, immune responses, antioxidant capacity, and growth performance in other aquatic species [55]. *Blautia*, which is known as an improvement in glucose and lipid homeostasis, was significantly upregulated by dietary CM in the CM4 group [56]. In this study, the lipid content of muscle significantly declined in CM4, which gradually descended with the increase of dietary CM levels. This data indicated that CM could lower lipid content and maintain intestinal flora homeostasis and disease resistance.

KEGG analysis revealed that the representative signaling pathways in CM1 and CM4 were associated with metabolism processes, including the PPAR signaling pathway and FoxO signaling pathway. PPARs constitute a subset of the ligand-activated nuclear hormone-receptor superfamily, encompassing three primary members: PPARα, PPARβ, and PPARγ. The PPARγ signaling pathway involved in this study partook in lipid metabolism, adipogenesis, and maintenance of metabolic homeostasis [57]. Prior research has demonstrated that PPARs expression is directly related to lipid metabolism in *Megalobrama amblycephala* fingerlings, also known as blunt snout bream [57]. FoxO represents a subfamily within the Fox (forkhead box) family, which comprises transcription factors characterized by a winged-helix configuration in the DNA-binding region [58]. The FoxO signaling pathway is implicated in various cellular physiological phenomena, including oxidative stress resistance, apoptosis, glucose metabolism, and longevity. A previous study has reported that *FOXO* played a critical role in maintaining hemolymph and intestinal microbiota homeostasis by promoting the expression of Relish, the transcription factor of the immune deficiency (IMD) pathway for expression of antimicrobial peptides (AMPs) in shrimp [59]. Thus, it may be speculated that CM may regulate muscle-lipid metabolism through activation of the PPARγ pathway and regulate the physiological role of intestinal microbiota homeostasis through the FoxO signaling pathway.

## 5. Conclusions

In conclusion, dietary supplementation with CM improved the antioxidative capability and muscle quality of Chinese soft-shelled turtles. Moreover, the supplementation of CM in the diet could enhance intestinal flora homeostasis. Our results also showed that metabolism-related pathways were upregulated when turtles were fed with dietary supplementation with CM. The appropriate dietary CM level for Chinese soft-shelled turtles was estimated to be 3.9 g/kg, based on the quadratic regression analysis of SOD and CAT. Therefore, this study can guide the application of CM as a functional feed additive to promote the health of turtles.

## Figures and Tables

**Table 1:** Growth performance of Chinese soft-shelled turtles fed diets containing different levels of CM for 6 weeks.

	CM0	CM0.5	CM1	CM2	CM4
^1^ I BW (g)	162.56 ± 4.50	159.61 ± 1.33	160.00 ± 3.15	158.89 ± 1.90	166.56 ± 0.97
^2^ FBW (g)	221.75 ± 6.35	223.31 ± 4.31	226.29 ± 1.66	227.51 ± 8.94	237.50 ± 4.45
^3^ WG (%)	37.53 ± 5.78	39.93 ± 3.05	42.00 ± 5.86	43.21 ± 5.67	44.59 ± 4.97
^4^ FCR	2.36 ± 0.11	2.26 ± 0.15	2.21 ± 0.06	2.30 ± 0.17	2.29 ± 0.08
^5^ SR	79.00 ± 2.31 ^a^	84.35 ± 1.61 ^a^	81.33 ± 2.83 ^a^	82.16 ± 2.66 ^a^	93.03 ± 2.67 ^b^

Data are presented as mean ± SD. Different letters in the same row represent a significant difference among groups (*p* < 0.05). ^1^ IBW (g) = initial mean weight; ^2^ FBW (g) = final mean weight; ^3^ WG (weight gain, %) = (FBW − IBW)/IBW × 100; ^4^ FCR (feed-conversion ratio) = (dry-feed weight per tank, g)/(total weight gain per tank, g); ^5^ SR (survival, %) = 100 × final amount of fish/initial amount of fish

**Table 2:** Muscle-proximate composition of Chinese soft-shelled turtles fed diets containing different levels of CM for 6 weeks.

	CM0	CM0.5	CM1	CM2	CM4
Moisture (%)	75.48 ± 5.12	73.77 ± 0.93	74.95 ± 0.97	74.23 ± 2.62	73.26 ± 2.00
Crude protein (%)	13.14 ± 0.79 ^a^	16.22 ± 0.20 ^b^	16.49 ± 0.12 ^b^	16.33 ± 1.26 ^b^	17.60 ± 0.30 ^c^
Crude lipid (%)	5.62 ± 0.78 ^b^	5.20 ± 1.04 ^b^	4.17 ± 0.65 ^a^	3.76 ± 0.36 ^a^	3.92 ± 0.32 ^a^
Ash (%)	5.40 ± 0.38	5.62 ± 1.32	5.43 ± 0.20	5.56 ± 0.19	5.44 ± 0.22

Data are presented as mean ± SD. Different letters in the same row represent a significant difference among groups (*p* < 0.05).

**Table 3:** Texture profiles of Chinese soft-shelled turtles fed diets containing different levels of CM for 6 weeks.

	CM0	CM0.5	CM1	CM2	CM4
Hardness (g)	957.75 ± 83.90 ^a^	997.25 ± 77.47 ^a^	1287.00 ± 120.53 ^b^	1251.25 ± 106.52 ^b^	1308.00 ± 115.00 ^b^
Springiness	0.41 ± 0.04	0.40 ± 0.03	0.38 ± 0.02	0.45 ± 0.04	0.45 ± 0.06
Cohesiveness	0.51 ± 0.08	0.55 ± 0.06	0.48 ± 0.06	0.60 ± 0.03	0.52 ± 0.05
Gumminess (g)	570.90 ± 67.75 ^a^	577.29 ± 43.22 ^a^	606.26 ± 64.67 ^ab^	661.98 ± 43.59 ^ab^	734.31 ± 78.18 ^b^
Chewiness (g)	178.12 ± 12.71 ^a^	181.09 ± 15.32 ^a^	219.43 ± 16.79 ^b^	220.47 ± 21.87 ^b^	184.49 ± 14.52 ^ab^
Resilience	0.26 ± 0.05	0.26 ± 0.05	0.26 ± 0.03	0.25 ± 0.04	0.25 ± 0.04

Data are presented as mean ± SD. Different letters in the same row represent a significant difference among groups (*p* < 0.05).

**Table 4:** Antioxidant enzyme activities in serum and liver of Chinese soft-shelled turtles fed with diets containing different CM levels for 6 weeks.

	CM0	CM0.5	CM1	CM2	CM4
Serum					
SOD (U/mgprot)	57.20 ± 1.88 ^a^	63.08 ± 2.54 ^b^	68.20 ± 3.51 ^b^	80.47 ± 2.03 ^c^	85.81 ± 2.36 ^c^
Liver					
CAT (U/mgprot)	11.80 ± 0.48 ^a^	11.37 ± 0.72 ^a^	15.43 ± 0.25 ^b^	14.99 ± 0.49 ^b^	16.99 ± 0.18 ^c^
T-AOC (U/mgprot)	2.87 ± 0.09 ^a^	2.95 ± 0.08 ^ab^	3.06 ± 0.04 ^b^	3.01 ± 0.08 ^ab^	3.06 ± 0.07 ^b^

Data are presented as mean ± SD. Different letters in the same row represent a significant difference among groups (*p* < 0.05).

## Data Availability

All data are available in the article and Supplementary Materials.

## Supplementary Materials

The following supporting information can be downloaded at: https://www.mdpi.com/article/10.3390/ani13162626/s1, Table S1: Composition and proximate analysis of experimental diets; Table S2: Serum biochemical indices of Chinese soft-shelled turtles fed with diets containing different curcumin levels for 6 weeks; Table S3: Primers used for qPCR; Table S4: Number of OTUs for each sample; Figure S1: Functional prediction of changed gut microbiota by KEGG; Figure S2: Top 10 significant KEGG pathways of up/down-regulated genes in CM0 vs CM1/CM4 group.
