# Supplementary material for: Dietary Curcumin Supplementation Could Improve Muscle Quality, Antioxidant Enzyme Activities and the Gut Microbiota Structure of Pelodiscus sinensis
Source: Animals (Basel). 2023 Aug 14;13(16):2626. doi: 10.3390/ani13162626 (PMC10451759; doi:10.3390/ani13162626)
Supplement: Supplementary file 1 [file animals-13-02626-s001.zip › animals-2538330-supplementary.pdf]

Table S1. Composition and proximate analysis of experimental diets. Data are given in g/kg (on dry weight basis)

	CM0	CM0.5	CM1	CM2	CM4
Ingredients (g/kg)					
White fish meal	400	400	400	400	400
Soybean meal	100	100	100	100	100
Soy protein concentration	100	100	100	100	100
Yeast meal	60	60	60	60	60
Fish oil	33	33	33	33	33
Soybean oil	33	33	33	33	33
α-starch	200	200	200	200	200
Zeolite powder	16.5	16.5	16.5	16.5	16.5
[1]Vitamin premix	5	5	5	5	5
[2]Mineral premix	20	20	20	20	20
Dicalcium phosphate	20	20	20	20	20
Choline chloride	2.5	2.5	2.5	2.5	2.5
Cellulose	10	9.5	9	8	6
Curcumin	0	0.5	1	2	4
Proximate composition (g/kg)					
Moisture	83.9	81.5	84.1	82.1	84.6
Crude protein	428.0	411.1	420.3	430.1	426.5
Crude lipid	91.9	93.6	88.8	87.4	89.1
Ash	144.6	150.7	136.7	145.9	140.9

[1]Vitamin mixture (mg/kg diet): thiamine hydrochloride, 5; riboflavin, 20; calcium pantothenate, 10; D-biotin, 0.6; pyridoxine hydrochloride, 10; folic acid, 1; inositol, 1000; L-ascorbyl-2-monophosphate-Mg, 60; α-tocopheryl acetate, 60; menadione, 5; nicotinic acid 120; retinol acetate, 5000 IU; cholecaliferol, 2000 IU.

[2]Mineral mixture (mg/kg diet): calcium dihydrogen phosphate monohydrate, 6000; five water hydroxy calcium propionate, 6540; ferrous sulphate hexahydrate, 42.5; magnesium sulphate heptahydrate, 1340; sodium dihydrogen phosphate, 1744; sodium chloride, 870; aluminium chloride hexahydrate, 3; potassium iodate, 2.5; potassium chloride, 1500; copper chloride dihydrate, 20; manganese sulphate monohydrate, 16; cobalt chloride hexahydrate, 20; zinc sulphate heptahydrate, 6.

Table S2. Serum biochemical indices of Chinese soft-shelled turtles fed with diets containing different curcumin levels for 6 weeks

	**CM0**	**CM0.5**	**CM1**	**CM2**	**CM4**
TP (g/L)	32.80 ± 1.57[a]	40.82 ± 0.39[b]	29.69 ± 2.06[a]	30.19 ± 0.81[a]	34.05 ± 1.04[a]
ALB (g/L)	7.20 ± 1.31	8.13 ± 0.90	6.33 ± 0.31	6.74 ± 0.58	8.19 ± 0.22
TG (mmol/L)	1.50 ± 0.14[a]	2.14 ± 0.05[ab]	2.14 ± 0.41[ab]	2.69 ± 0.28[b]	3.05 ± 0.45[b]
TCHO (mmol/L)	5.54 ± 0.48[a]	6.98 ± 0.27[b]	7.14 ± 0.24[b]	7.11 ± 0.12[b]	7.59 ± 0.11[b]
GLU (mmol/L)	3.28 ± 1.08	3.67 ± 0.44	4.02 ± 0.63	4.64 ± 0.30	4.98 ± 0.65
ALP (U/L)	1180.33 ± 207.55[b]	1234.33 ± 266.68[b]	1146 ± 120.09[b]	857.67 ± 77.12[a]	832.33 ± 59.50[a]
AST (U/L)	119.00 ± 7.51[c]	122.67 ± 9.96[c]	93.67 ± 4.70[b]	63.33 ± 2.85[a]	64.33 ± 2.40[a]

TP, total protein; ALB, albumin; TG, triglyceride; TCHO, total cholesterol; GLU, glucose; ALP, alkaline phosphatase; AST, aspartate transaminase.

Data were presented as mean ± SD. Different letters in the same row represent a significant difference among groups ($P < 0.05$).

Table S3. Primers used for qPCR

Gene ID	Gene symbol	Forward primer (5'-3')	Reverse primer (5'-3')
XM_006126482.3	*SLC38A1*	TCGGCATGGTACGTGGATGGT	GGCTCCAGTCCAGGGCAGATAA
XM_025179876.1	*S100B*	TGGCGATGCTGAATGCGACTTC	GGTGGCTTCTCCGATGCTTCAC
XM_025190937.1	*OVCH1*	CCAGACGTGTTGTCGGAGGAGA	ACTGATGACGACGCCACCACA
XM_006135326.3	*AGTR2*	GTCTCCCTGCCCGCTTCTGTAT	ACCGCCACCACCACACTGTT
XM_006112915	*β-actin*	TGATGGACTCAGGTGACGGTGT	GGCTGTGGTGGTGAAGCTGTAG

Table S4. Number of OTUs for each sample

ID	domain	phylum	class	order	family	genus	species
CM0_1_1	9	2	10	28	87	110	45
CM0_1_2	12	2	3	58	91	84	40
CM0_1_3	3	0	3	15	75	43	22
CM0_1_4	14	4	30	61	149	206	59
CM0_2_1	8	3	10	44	102	123	58
CM0_2_2	21	7	36	52	179	243	80
CM0_3_1	8	3	16	67	121	150	47
CM0_4_1	7	6	43	46	168	253	72
CM1_1_1	16	3	9	59	152	132	58
CM1_1_2	10	3	29	51	273	195	88
CM1_2_1	3	3	6	14	60	64	35
CM1_2_2	19	1	16	62	135	144	47
CM1_2_3	12	3	5	46	75	89	26
CM1_3_1	13	3	21	47	193	198	92
CM1_3_2	4	0	5	10	82	66	48
CM1_3_3	4	0	4	20	76	66	31
CM1_4_1	1	5	13	27	104	138	63
CM4_1_1	3	6	27	167	355	334	134
CM4_2_1	2	3	7	30	84	84	57
CM4_2_2	26	3	38	53	184	252	81
CM4_2_3	30	5	41	47	144	234	88
CM4_2_4	12	2	25	28	125	207	76
CM4_3_1	5	1	4	9	52	57	32
CM4_4_1	17	3	31	50	162	261	74

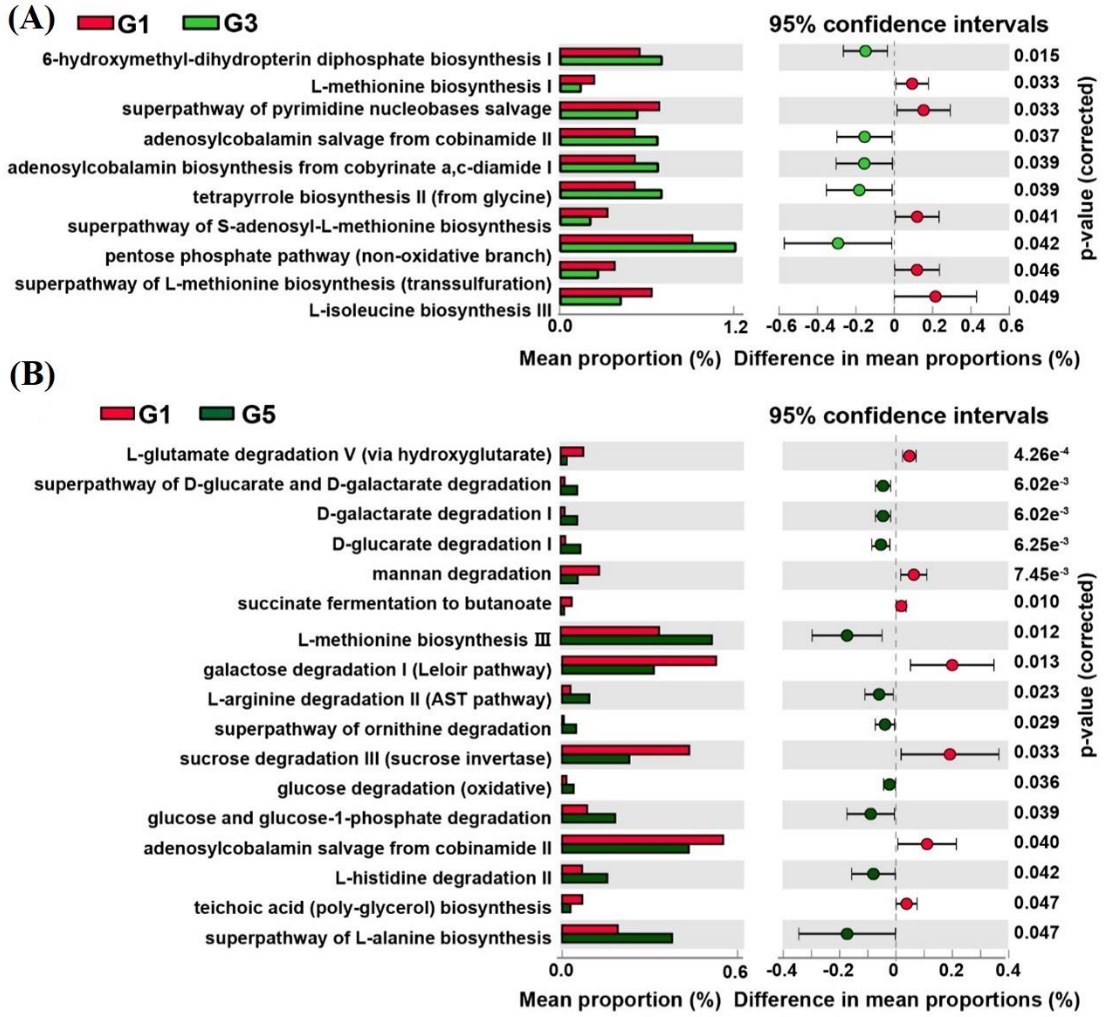

Figure S1. Functional prediction of changed gut microbiota by KEGG. (A) CM1 vs. CM0; (B) CM4 vs. CM0. Differences were considered significant at $P < 0.05$ using t-test.

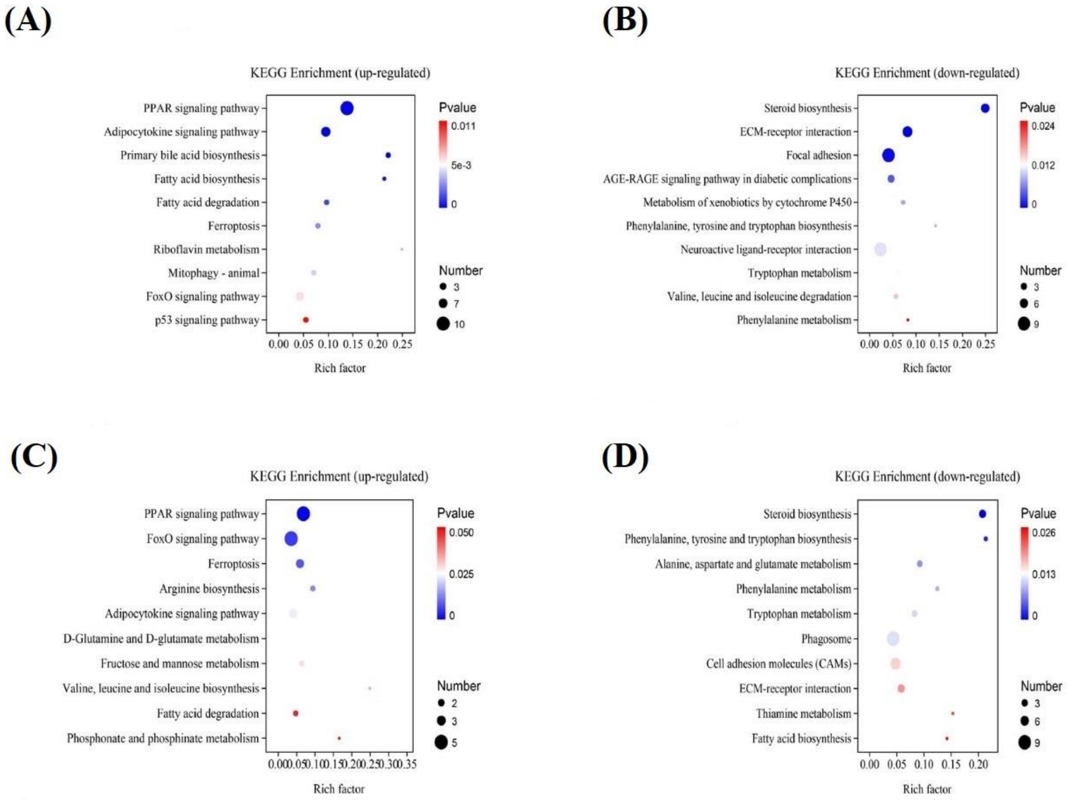

Figure S2. (A) Top 10 significant KEGG pathways of up-regulated genes in CM0 vs CM1 group ($P < 0.05$). (B) Top 10 significant KEGG pathways of down-regulated genes in CM0 vs CM1 group ($P < 0.05$). (C) Top 10 significant KEGG pathways of up-regulated genes in CM0 vs CM4 group ($P < 0.05$). (D) Top 10 significant KEGG pathways of down-regulated genes in CM0 vs CM4 group ($P < 0.05$)
